# Disrupted Brain Intrinsic Networks and Executive Dysfunction in Cirrhotic Patients without Overt Hepatic Encephalopathy

**DOI:** 10.3389/fneur.2018.00014

**Published:** 2018-01-25

**Authors:** Zhe-Ting Yang, Hua-Jun Chen, Qiu-Feng Chen, Hailong Lin

**Affiliations:** ^1^Department of Radiology, Fujian Medical University Union Hospital, Fuzhou, China; ^2^College of Computer and Information Sciences, Fujian Agriculture and Forestry University, Fuzhou, China

**Keywords:** cirrhosis, executive function, executive control network, default mode network, salience network

## Abstract

**Objective:**

Patients with cirrhosis often exhibit cognitive deficits, particularly executive dysfunction, which is considered a predictor of overt hepatic encephalopathy (OHE). We examined brain intrinsic networks associated with executive function to investigate the neural basis of this cognitive deficiency in cirrhosis.

**Methods:**

Resting-state functional MRI data were acquired from 20 cirrhotic patients and 18 healthy controls. Seed-based correlation analysis was used to identify the three well-known networks associated with executive function, including executive control (ECN), default mode (DMN), and salience (SN) networks. Functional connectivity (FC) within each network was compared between groups and correlated with patient executive performance (assessed by the Stroop task).

**Results:**

Patients showed decreased FC between the ECN seed (right dorsolateral prefrontal cortex) and several regions (including right middle/inferior frontal gyrus, left inferior frontal gyrus, bilateral inferior/superior parietal lobules, bilateral middle/inferior temporal gyrus, and right medial frontal gyrus), between the DMN seed [posterior cingulate cortex (PCC)] and several regions (including bilateral medial frontal gyrus, bilateral anterior cingulate cortex, bilateral superior frontal gyrus, bilateral precuneus/PCC, left supramarginal gyrus, and left middle temporal gyrus), and between the SN seed (right anterior insula) and right supramarginal gyrus. FC strength in the ECN and SN was negatively correlated with patient performance during the Stroop task.

**Conclusion:**

Disrupted functional integration in the core brain cognitive networks, which is reflected by reductions in FC, occurs before OHE bouts and may play an important role in the neural mechanism of executive dysfunction associated with cirrhosis.

## Introduction

Overt hepatic encephalopathy (OHE), which is one of the most serious complications of liver cirrhosis, severely affects the lives of patients and is associated with poor survival (1, 2). Therefore, as a predictor of OHE, mild cognitive impairment associated with cirrhosis has drawn increasing attention (2, 3). Of these cognitive impairments, executive dysfunction, such as deficits in set switching, cognitive flexibility, and interference inhibition, is considered to be an early and key component (4, 5). Several studies have demonstrated usefulness of executive function examination in detecting the initial stage of hepatic encephalopathy (HE). Thus, clinicians and researchers have begun exploring the neural mechanisms of executive deficit in patients with hepatic cirrhosis (4, 6, 7).

Neuropsychological findings have demonstrated that executive control deficiency in cirrhotic patients is associated with dysfunction of the anterior attention system, whose function is modulated by the prefrontal cortex, anterior cingulate cortex (ACC), supplemental motor cortex, and basal ganglia (4). Weissenborn et al. (8, 9) proposed that executive dysfunction in cirrhosis contributes to an alteration of energy metabolism in the medial and lateral frontal cortex, anterior cingulate gyrus, and parietal structures. In addition, neuroimaging studies using task-based functional magnetic resonance imaging (fMRI) have also revealed less activation in the brain network that involves the ACC, prefrontal cortex, parietal lobe, and temporal fusiform gyrus when cirrhotic patients without OHE performed the Stroop task (7) [a test assessing executive function that requires a number of cognitive abilities to occur in tandem, such as selective attention, dual processing, processing speed, and cognitive flexibility (10)].

Recent task-free (resting-state) fMRI studies in patients with cirrhosis demonstrated altered functional connectivity (FC) within the distinct brain intrinsic networks [such as the executive control network (ECN), default mode network (DMN), and salience network (SN) (11–13)] that may underlie abnormal executive function. Existing studies, however, cannot provide direct evidence regarding the relationship between this cognitive deficiency and changes in brain network FC, because they have not conducted specific neuropsychological tests to assess patient executive function. Furthermore, these resting-state fMRI studies yielded inconsistent results concerning FC in patients. For example, Qi et al. (14) reported both decreased and increased FC associated with cirrhosis within the DMN, but other studies only found a reduction in FC in the same network (13, 15).

In this study, we explored alterations of network-level neural function using resting-state fMRI in cirrhotic patients and examined associations between these alterations of intrinsic neural networks and patient executive dysfunction as assessed by the Stroop task. The current study will provide new insights into the neural mechanisms of executive function deficit in cirrhotic patients from the perspective of resting-state brain function.

## Subjects and Methods

### Participants

This study was approved by the Research Ethics Committee of the Fujian Medical University Union Hospital, China. Written informed consent was obtained from each participant prior to the study. Twenty cirrhotic patients without overt HE and 18 healthy controls were included. Table 1 provides demographic and clinical characteristics of the study participants. No significant differences were detected in age, gender, or education level between the two groups. Hepatic cirrhosis was diagnosed based on clinical, laboratory, imaging (CT, MR, and ultrasound examination), and, in some (5/20), histological features. The Child–Pugh score was used to assess functional status of each patient. All patients underwent a detailed neurological examination to confirm absence of overt HE. Manifestation of overt HE refers to the West Haven Criteria. The exclusion criteria were as follows: (1) diagnosis of current overt HE or other neuropsychiatric disorder, (2) taking psychotropic medications, (3) diagnosis of uncontrolled endocrine or metabolic disease (e.g., thyroid dysfunction), (4) alcohol abuse within 6 months prior to the study, (5) contraindications for MRI, and (6) head movement greater than 2-mm maximum displacement in any of the *x, y*, or *z* directions or greater than 2° angular rotation in any axis during resting-state fMRI examination (16, 17).

**Table 1:** Demographic and clinical characteristics of the subjects.

	Healthy controls (*n* = 18)	Cirrhotic patients (*n* = 20)	*P* value
Age (years)	50.4 ± 7.2	51.2 ± 9.2	0.794
Sex (male/female)	13/5	16/4	0.709 (χ^2^-test)
Education level (years)	9.0 ± 2.3	8.7 ± 2.8	0.682
Etiology of cirrhosis (Hepatitis B virus/alcoholism/Hepatitis B virus and alcoholism/other)	–	12/3/2/3	–
Child–Pugh stage (A/B/C)	–	3/12/5	–
EncephalApp_Stroop test			
Offtime (seconds)	91.3 ± 25.3	125.3 ± 30.8	0.001
Number of runs for “Off” state	6 (5–9)	8 (5–14)	0.006
Ontime (seconds)	103.5 ± 28.2	146.4 ± 39.9	0.001
Number of runs for “On” state	6 (5–11)	8 (5–17)	0.022
Offtime + ontime (seconds)	194.8 ± 52.9	271.7 ± 69.4	0.001
Ontime − offtime (seconds)	12.2 ± 7.9	21.2 ± 16.0	0.034

### Stroop Task Performance

The Stroop color-word task was employed to assess executive function. This test has been used widely in cirrhotic patients (4, 7, 18–20). Subjects performed a Stroop color-word paradigm using a Stroop smartphone application (app) called EncephalApp_Stroop.^1^ This app (Chinese version) was downloaded from the Apple app Store and used on the Apple iPod platform. EncephalApp_Stroop, which tests psychomotor speed and cognitive flexibility, was proposed by Bajaj and his colleagues (20) and has been validated in characterizing early cognitive deficits (e.g., executive dysfunction) among cirrhotic patients (18, 19). There are two components (i.e., “Off” and “On” states) in the task, depending on whether the stimuli are congruent or incongruent. In the easier “Off” state, subjects were presented with neutral stimulus-pound signs (“#”) in red, green, or blue. Subjects were asked to identify the color as quickly and accurately as possible by pressing a button. In the subsequent “On” state, subjects were presented with incongruent stimuli (e.g., the word “blue” printed in a red font), and subjects were asked to identify the font color while suppressing automatic word reading. During both “Off” and “On” states, if the subject made a mistake, then the run stopped and restarted.

The EncephalApp_Stroop outcomes included the following: (1) total time to complete five correct runs in the “Off” state (Offtime) and five correct runs in the “On” state (Ontime) and (2) number of runs needed to complete five correct “Off” runs and five correct “On” runs. Offtime primarily assesses psychomotor speed, while Ontime measures response inhibition and psychomotor speed. In addition, the Ontime minus Offtime variable was calculated to control for psychomotor speed and was regarded as a measure of cognitive flexibility. The number of runs needed to make five correct runs indicated the number of errors during the task, which reflected processing accuracy.

### MRI Data Acquisition

MRI data were acquired using a 3-T MR scanner (Siemens, Verio, Germany). All subjects were instructed to keep still, keep their eyes closed but not to fall asleep, and not to engage in any specific thinking activities during data acquisition. The 180 resting-state fMRI volumes were acquired using an echo planar imaging sequence, and the parameters were as follows: repetition time (TR) = 2,000 ms, echo time (TE) = 25 ms, flip angle = 90°, field of view (FOV) = 240 mm × 240 mm, matrix = 64 × 64, and slice thickness = 4 mm with no gap. Each brain volume included 35 axial slices. High-resolution T1-weighted images were acquired with a magnetization-prepared rapid gradient echo sequence, and the parameters were as follows: TR = 1,900 ms, TE = 2.48 ms, flip angle = 9°, FOV = 256 mm × 256 mm, matrix = 256 × 256, number of sagittal slices = 176, and slice thickness = 1 mm. T1-weighted anatomic images were used for spatial normalization of functional images.

### fMRI Data Preprocessing

We utilized a Data Processing & Analysis for Brain Imaging toolbox (DPABI^2^), which was based on statistical parametric mapping^3^ and the Resting-State fMRI Data Analysis Toolkit (REST^4^) for fMRI data analysis. The preprocessing procedure was as follows. The first 10 time points were discarded. Slice timing and head motion correction were then performed. According to the criterion of a displacement >2 mm or an angular rotation >2° in any direction, none of the participants were excluded. The summary scalars of gross head motion were matched between healthy control and patient groups (all *P* > 0.25): mean translational movement in *x, y*, and *z* directions were 0.068 ± 0.057 vs 0.069 ± 0.060 mm, 0.093 ± 0.062 vs 0.094 ± 0.059 mm, and 0.209 ± 0.114 vs 0.245 ± 0.196 mm; mean rotational motion in three axes were 0.229° ± 0.193° vs 0.205° ± 0.159°, 0.113° ± 0.083° vs 0.151° ± 0.116°, and 0.093° ± 0. 093° vs 0.086 ± 0.076°. Also, we calculated frame-wise displacement (FD) index, which measures volume-to-volume changes in head position (21, 22). The mean FD in healthy control and patient groups were 0.121 ± 0.044 and 0.148 ± 0.091, respectively. One-sample *t*-test showed that they were significantly less than 0.2 mm (*P* < 0.05); and two-sample *t*-test showed no significant difference in FD between two groups (*P* = 0.168). Subsequently, the resulting data were normalized to Montreal Neurological Institute (MNI) space and resampled to 3-mm isotropic voxels. After the images were smoothed with a 4-mm full width half maximum Gaussian kernel, the linear trend of time courses was removed, and band-pass temporal filtering (0.01–0.08 Hz) was performed. Finally, the six head motion parameters as well as white matter, cerebrospinal fluid, and global brain signals were regressed out as nuisance variables. Whether preprocessing should include global signal regression (GSR) is controversial (23, 24); thus, we also conducted an analysis without the GSR, and the results were shown in the supplementary materials (Figures S1–S3 in Supplementary Material). Based on the analyses with and without the GSR, we investigated the patterns of between-group FC difference in the ECN, DMN, and SN; and we found that they were very similar to each other.

### Functional Connectivity

Functional connectivity was calculated using seed-based correlation analysis. We examined FC associated with three well-described neural networks, ECN, DMN, and SN, because these networks have been shown to be involved in executive processes (10, 25–28). Each network was defined by the voxel-wise Pearson correlation with a reference time series extracted as the simple average time series of all voxels within a 6-mm spherical seed. Networks and corresponding seed ROI were as follows. The ECN seed was placed in the right dorsolateral prefrontal cortex (RDLPFC, MNI: 44, 36, 20). The DMN seed was located in the posterior cingulate cortex (PCC, MNI: 1, −55, 17). The SN was defined by a seed in the right anterior insula (RAI, MNI: 38, 26, −10). Pearson correlation maps were then normalized using a Fisher *Z*-transform.

The statistical module of the DPABI^5^ was applied for resting-state fMRI data analysis. For each group, the random-effect one-sample *t*-test was used to determine the spatial distribution pattern of FC of each seed. A threshold of *P* < 0.001 [false discovery rate (FDR) corrected] was set to identify significance level. For each subject, the mean FC strength (denoted by averaged *Z* score) of each network was then calculated and compared between the two groups using independent sample Student’s *t*-tests.

Voxel-wise comparisons of FC between the two groups using a two-sample t-test were then performed with an explicit mask from the union set of the one-sample t-test results of the two groups. The significance threshold of between-group differences was set to *P* < 0.05 (FDR corrected) and cluster size >270 mm^3^.

### Brain–Behavior Correlation Analysis

Globally, we examined correlations between mean FC strength of each network and results of the Stroop task using Spearman correlation analysis (*P* < 0.05) in cirrhotic patients. Locally, a voxel-wise correlation analysis was conducted to determine the regions in which FC was significantly correlated with patients’ performances during the Stroop task. The statistical threshold was set at *P* < 0.01 and cluster size > 243 mm^3^, which corresponded to a corrected *P* < 0.05. This correction was determined by Monte Carlo simulations using the AFNI Alphasim program and confined to the areas with between-group FC differences.

## Results

Both healthy controls and cirrhotic patients performed faster and showed higher accuracy in the congruent task (“Off” state) than in the incongruent task (“On” state). During both “Off” and “On” states in the EncephalApp_Stroop test, the cirrhotic patients performed more poorly than the healthy controls. The patients needed more time and additional runs to complete the five correct “Off” runs and five correct “On” runs (Table 1). In addition, the Ontime minus Offtime variable significantly increased in the patient group. These results indicate slower psychomotor speed and impaired cognitive flexibility in the cirrhotic patients.

**Figure 1:**
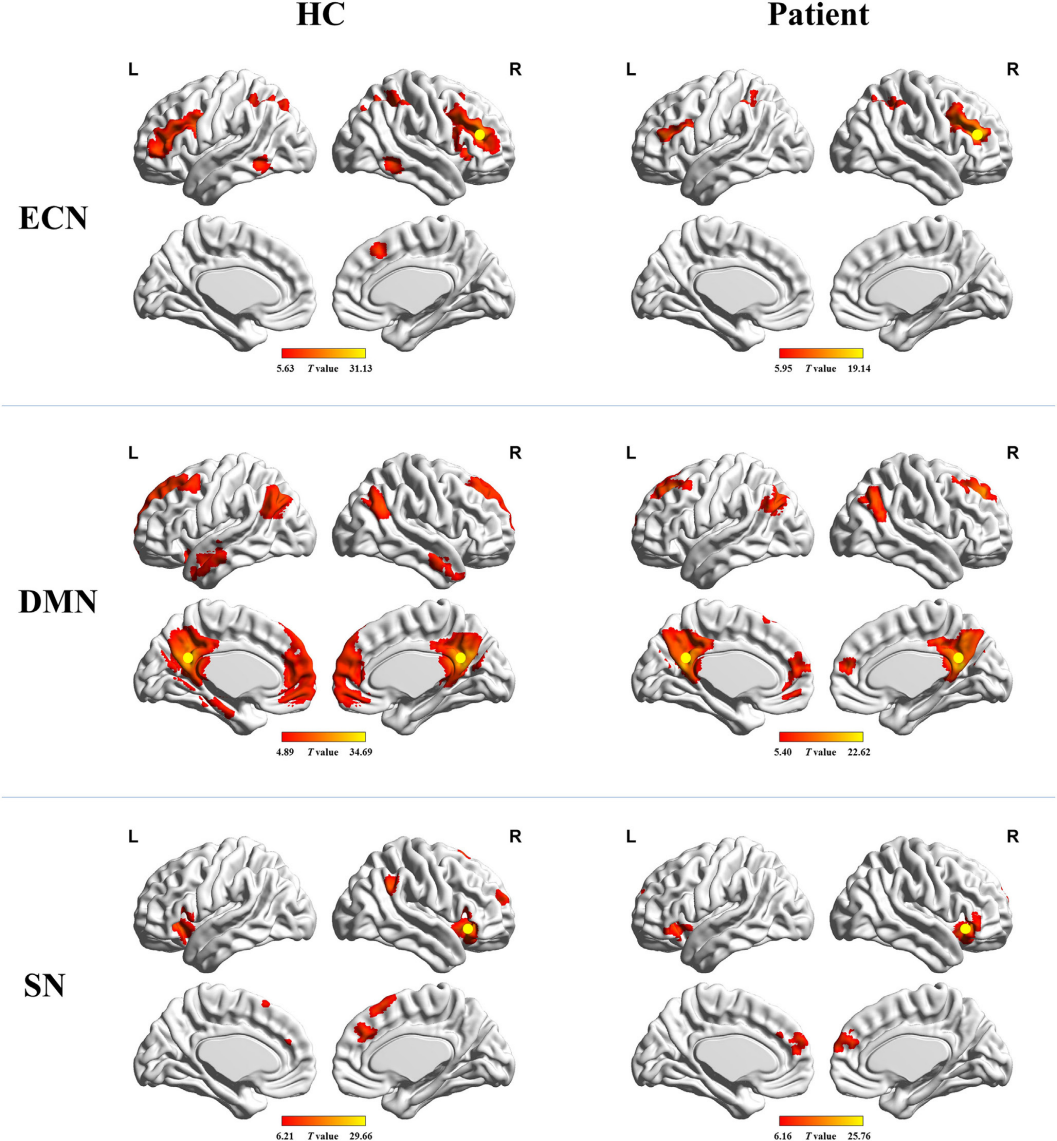
Functional connectivity maps for healthy controls and cirrhotic patients. Yellow circles denote locations of the seed regions used to define each network. Abbreviations: ECN, executive control network; DMN, default mode network; SN, salience network. A threshold of *P* < 0.001 (false discovery rate corrected) was set to identify significance level.

Figure 1 shows the FC maps for the ECN, DMN, and SN. In the healthy controls, the ECN mainly included the bilateral DLPFC and posterior parietal cortex. The DMN primarily comprised the bilateral PCC/precuneus, medial prefrontal cortex (MPFC), lateral parietal cortex, anterior temporal lobes, and hippocampi. The dorsal ACC/MPFC and the bilateral anterior insula (AI) formed components of the SN. These components and locations for the ECN, DMN, and SN were consistent with previous studies (10, 25–28). The patient group showed a FC pattern similar to that of the healthy group but less extensive (visual inspection).

**Figure 2:**
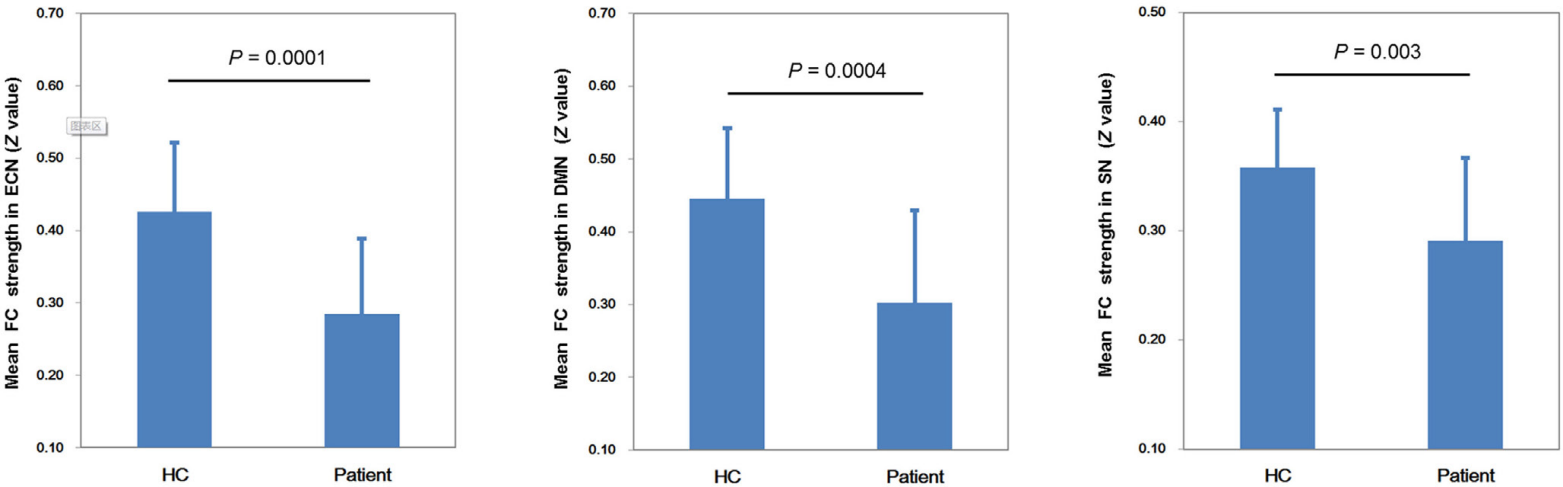
Mean network connectivity strengths for the two groups. The cirrhotic patients showed significant reductions of mean functional connectivity (FC) strength within the executive control network (ECN), default mode network (DMN), and salience network (SN).

**Figure 3:**
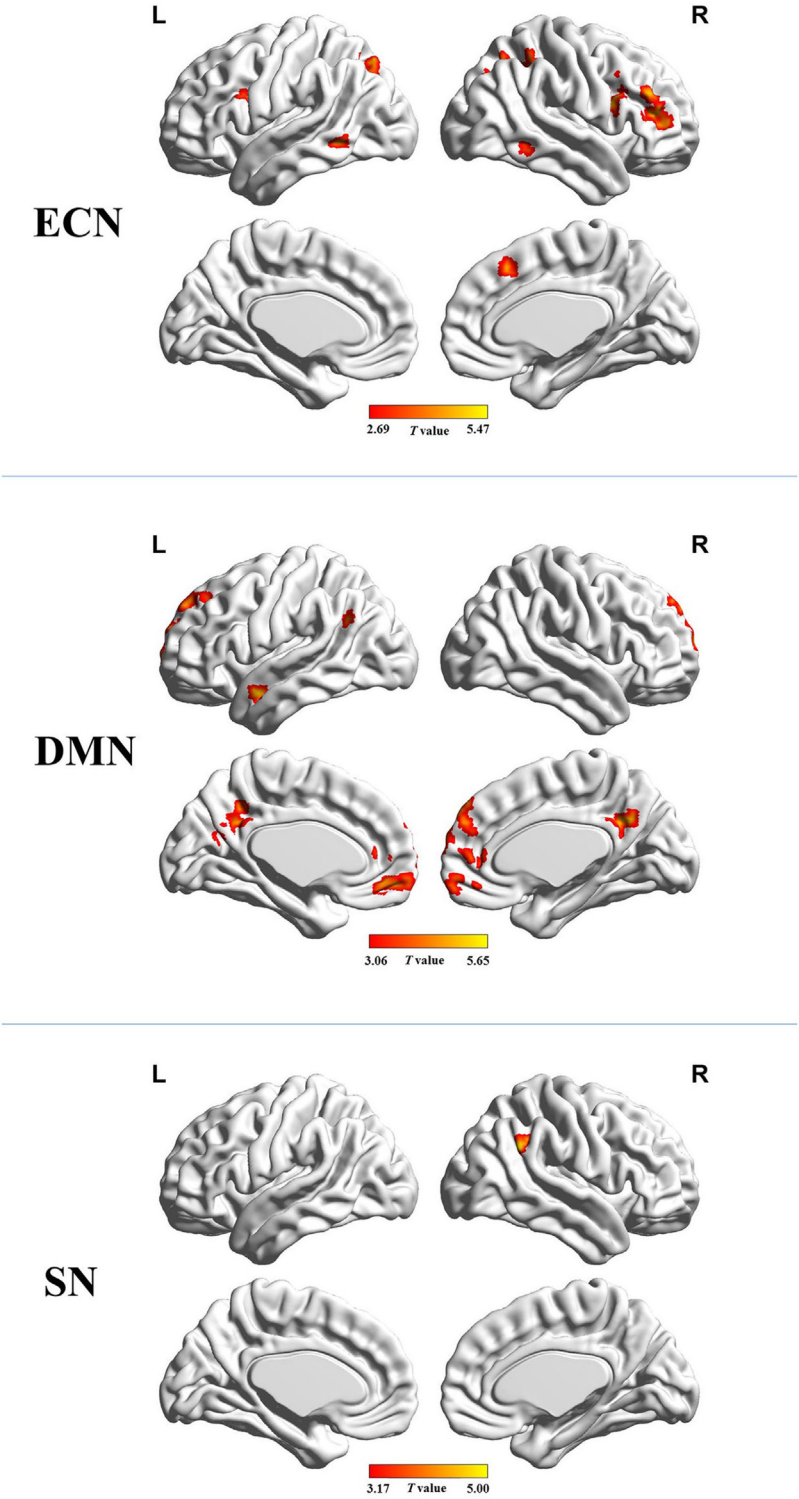
Brain regions in which functional connectivity with the network seed was significantly reduced in the cirrhotic patients. Abbreviations: ECN, executive control network; DMN, default mode network; SN, salience network. A threshold of *P* < 0.05 (false discovery rate corrected) was set to identify significance level.

Figure 2 shows the mean network connectivity strengths for the two groups. Globally, the cirrhotic patients had significantly decreased connectivity in all three networks when compared with healthy controls. Locally, voxel-wise analysis revealed several brain regions in which FC with the network seed was significantly reduced in cirrhotic patients (Figure 3 and Table 2): (1) for the ECN, decreased connectivity with the RDLPFC seed was found in the right middle/inferior frontal gyrus, left inferior frontal gyrus, bilateral inferior/superior parietal lobules, bilateral middle/inferior temporal gyrus, and right medial frontal gyrus; (2) for the DMN, reduced connectivity with the PCC seed was detected in the bilateral medial frontal gyrus, bilateral ACC, bilateral superior frontal gyrus, bilateral precuneus/PCC, left supramarginal gyrus, and left middle temporal gyrus; (3) for the SN, FC between the right supramarginal gyrus and RAI seed was decreased. No significant increase in FC was detected in any network.

**Table 2:** Significant reduction of functional connectivity in patients with cirrhosis.

Seed	Connected area	Voxels	Brodmann area	Montreal Neurological Institute coordinates			Peak *T*-value
				*x*	*y*	*z*	
RDLPFC in ECN	Right middle frontal gyrus	32	46	51	36	27	5.47
	Right middle/inferior frontal gyrus	83	46/10	45	48	9	4.97
	Left inferior/middle temporal gyrus	28	20/21	−48	−48	−9	4.37
	Right inferior/middle frontal gyrus	76	9/8	51	12	39	4.29
	Left inferior frontal gyrus	28	9	−48	3	18	4.07
	Right inferior/superior parietal lobule	55	7/40/39	33	−57	42	4.06
	Right middle/inferior temporal gyrus	16	21/20	51	−45	−15	3.97
	Right inferior parietal lobule	23	40	42	−45	45	3.96
	Left inferior/superior parietal lobule	22	7	−24	−75	51	3.82
	Right medial frontal gyrus	12	8	6	24	48	3.65
PCC in DMN	Left middle temporal gyrus	15	21	−51	6	−18	5.65
	Bilateral medial frontal gyrus	54	11	−6	48	−9	4.65
	Right precuneus/PCC	35	31/7	9	−51	30	4.59
	Bilateral anterior cingulate cortex	41	32/10	0	45	9	4.43
	Left superior frontal gyrus	12	10	−12	69	6	4.37
	Left supramarginal gyrus	12	40	−48	−60	30	4.36
	Left precuneus/PCC	62	31/7	−9	−51	42	4.25
	Left superior frontal gyrus	16	9	−15	51	42	3.97
	Right superior frontal gyrus	19	10	12	69	15	3.87
	Right superior frontal gyrus	30	9	15	57	39	3.83
	Left superior frontal gyrus	10	9	−18	36	42	3.78
	Right superior frontal gyrus	11	10	12	54	27	3.36
RAI in SN	Right supramarginal gyrus	34	40	60	−51	30	5.00

*RDLPFC, right dorsolateral prefrontal cortex; ECN, executive control network; PCC, posterior cingulate cortex; DMN, default mode network; RAI, right anterior insula; SN, salience network*.

**Figure 4:**
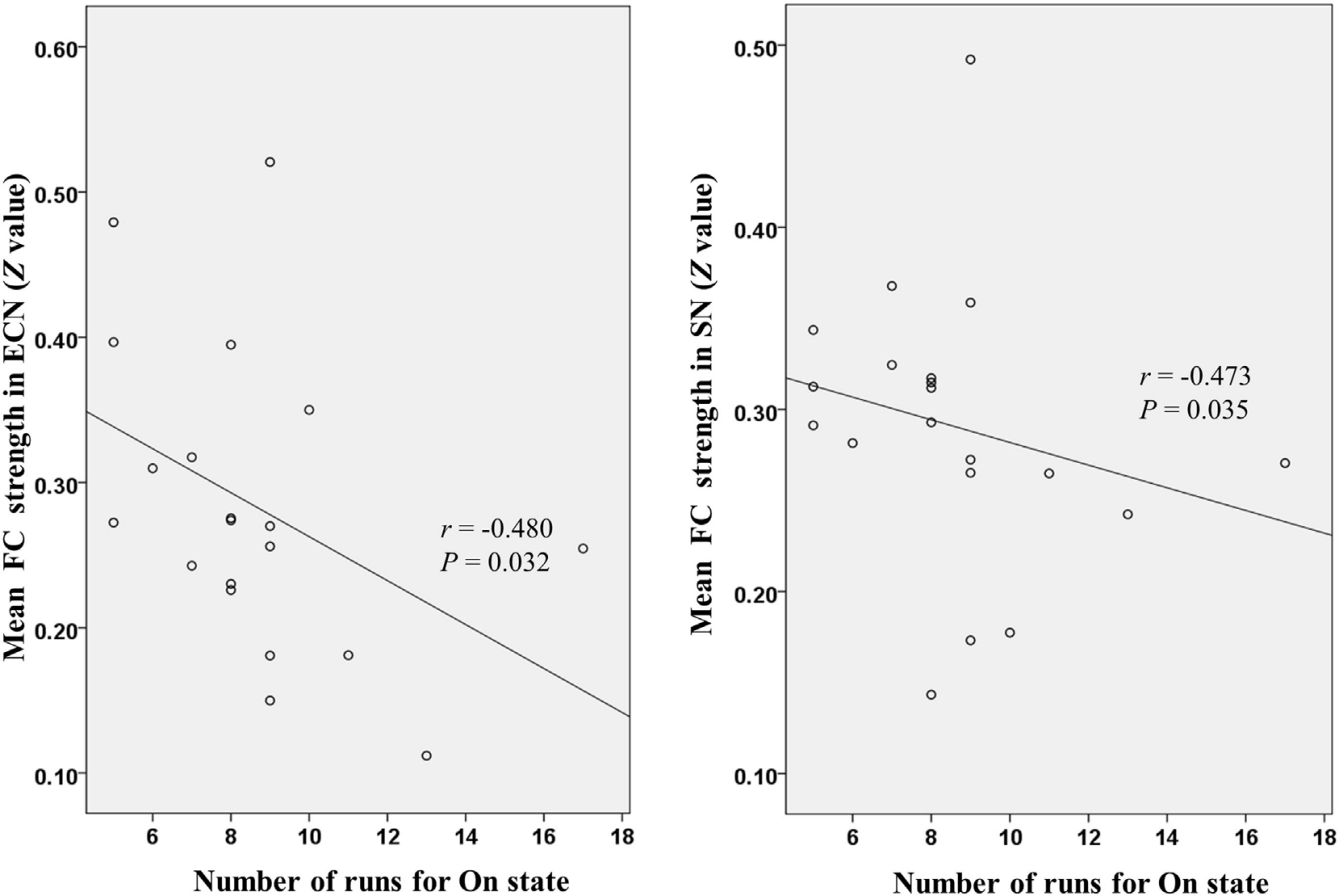
Scatter plots depicting correlations between Stroop task results (i.e., number of runs for the “On” state) and mean functional connectivity (FC) strength within the executive control network (ECN) and salience network (SN) in the patient group.

**Figure 5:**
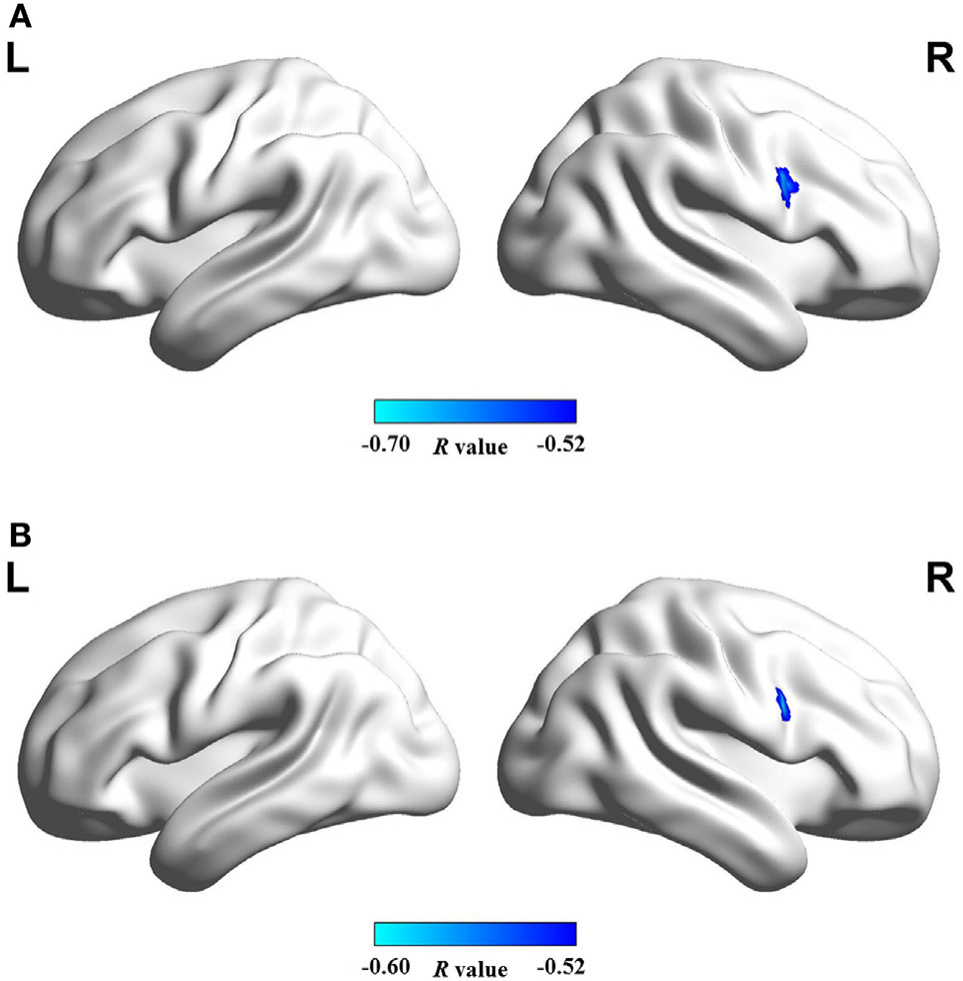
Brain regions in which functional connectivity with ECN RDLPFC seed was significantly correlated with the cirrhotic patients’ performances in Stroop task, i.e., **(A)** ontime and **(B)** ontime minus offtime. Abbreviations: ECN, executive control network; RDLPFC, right dorsolateral prefrontal cortex.

Figure 4 shows that mean network connectivity strengths of the ECN and SN were significantly correlated with Stroop task results (i.e., number of runs for the “On” state) in the patient group. Furthermore, voxel-wise analysis revealed that connectivity between the right inferior/middle frontal gyrus and the ECN DLPFC seed was significantly correlated with two measures of the EncephalApp_Stroop test (i.e., Ontime and Ontime minus Offtime) (Figure 5). Several correlations between FC within the DMN (e.g., between the PCC seed and MPFC) and Stroop task performance approached but did not reach significance after the multiple correction procedure.

## Discussion

In the current study, we examined brain intrinsic networks associated with executive function in cirrhotic patients and healthy controls. Significant FC reduction within three core cognitive networks (i.e., ECN, DMN, and SN) was identified in the patient group, which further supports the hypothesis that the metabolic dysfunction induced by cirrhosis contributes to disruption of brain networks (8, 9, 13, 29, 30). In addition, the neuropsychological test based on the Stroop task was conducted to assess executive function of subjects. Consistent with previous reports (4, 7, 20), the patients performed significantly worse as reflected by the longer time with more errors to complete the Stroop task. These results indicate slower psychomotor speed and impaired cognitive flexibility associated with cirrhosis, which could consequently lead to executive dysfunction (especially deficits in executive control). Furthermore, we found a correlation between impaired executive performance and disrupted FC in the ECN and SN, which provides the favorable evidence that abnormal functional integration of intrinsic brain networks plays an important role in the neural basis of this cognitive deficiency in cirrhosis.

Resting-state fMRI studies based on the data-driven method (e.g., independent component analysis, ICA) have demonstrated decreased FC in the core cognitive brain networks of cirrhotic patients, including the ECN (11, 12), DMN (13, 15), and SN (11, 12). Consistent with these reports, our findings derived from a hypothesis-driven approach further validate that disruption of functional integration is the major change in the intrinsic brain network, which precedes overt manifestations of HE. This network disconnectivity may be attributed to the following cirrhosis-induced abnormalities: (1) a disturbance in brain tissue energy metabolism given that many regions with decreased glucose uptake, as revealed by positron emission tomography, involve the three network areas described above (8, 9, 31); (2) an alteration in cerebral blood flow (32, 33), considering the tight coupling between brain blood supply and FC strength (34); and (3) damage to white matter fibers such as reduced axonal integrity and demyelination (35) given the fundamental role of fibers in connecting the distinct regions within the intrinsic brain networks (36). Taken together, our results provide further evidence of cirrhosis-related disruption of FC prior to the OHE episode.

Many studies have identified a critical role of the DLPFC in dynamic tuning of the executive control process, especially in conflict resolution (27, 37–39). Recent studies further emphasize the importance of intact functioning of the whole ECN (whose pivotal site is the DLPFC) for adequate cognitive control (10, 40). Deficiencies in synchronized connectivity among ECN regions are accompanied by altered executive control functioning (25, 41). Thus, it is predictable that FC reduction in cirrhotic patients correlates with impaired performance in the Stroop test (a task purported to require resolution of interference).

Functional disconnection in the DMN and its association with cognitive impairments among cirrhotic patients without OHE have been well documented. For example, an ICA study (13) revealed a correlation between cirrhosis-related FC reduction in the precuneus/PCC and poor performance in the Digit Symbol Test, which assesses psychomotor speed, attention, and visual memory, as well as in the Block Design Test, which tests visuomotor coordination, visuospatial reasoning, praxis, and psychomotor speed (42). However, in this study, in examining the correlation between FC within the DMN and Stroop task performance, some correlations approached but did not reach statistical significance. This may be due to the small sample size and limitations of the fMRI data analysis method (i.e., the seed-based connectivity approach that primarily examines the FC pattern of the network pivotal seed).

The bilateral AI and ACC, anchored within the SN, are responsible for modulating saliency detection, attentional capture enhanced by error signals, and dynamic cognitive control (26, 43). Recent studies further demonstrate that the SN engages in initiating control signals that activate the ECN and deactivate the DMN (26, 44). Thus, appropriate cognitive control abilities rely on intact SN functioning. SN dysfunction has been found to be associated with patient Stroop performance in cases of neuropsychological disease (28). Similarly, our results indicate a correlation between altered FC in the SN and error number when the cirrhotic patients performed the Stroop task with incongruent stimuli.

Several limitations of this study should be noted. First, the etiology of cirrhosis was heterogeneous in the patients. Given that the distinct etiologies could lead to the differences in behavioral and cerebral structural changes in patients (45, 46), future studies should examine altered FC in intrinsic brain networks and its relationship with executive function changes in patients with specific cirrhosis etiologies. Second, physiological noise should be considered in resting-state FC analysis. In this study, we could not eliminate completely cardiac and respiratory fluctuations through temporal filtering (band-pass 0.01–0.08 Hz). Third, we evaluated executive function using the Stroop task, which primarily measures executive control abilities, reflecting success in detecting and resolving cognitive conflict. Additional neuropsychological tests, such as the Wisconsin Card Sorting Test that evaluates several domains of executive function, including strategic planning, cognitive shifting, and perseveration (47), are recommended to assess other aspects of executive function so that the relationship between executive dysfunction and disrupted brain network FC can be investigated more comprehensively. Fourth, the multiple comparisons correction was not performed when we explored the correlation between patients’ performance in the Stroop task and the mean FC strength within ECN and SN. Thereby, this finding was preliminary. Future studies with larger sample size are required to validate the relevant result.

In conclusion, based on combined analyses of FC in three large-scale neural networks, this study demonstrated disrupted functional integration in cirrhotic patients. Moreover, our assessment of brain–behavior relationships sheds light on the important role of functional disconnection of brain networks in executive dysfunction associated with cirrhosis. Taken together, these findings suggest that reduced FC in key brain networks may represent an important feature in understanding and treating executive dysfunction in cirrhotic patients before OHE.

## Author Contributions

H-JC and Z-TY conceived and designed the study, acquired and analyzed the data, and wrote the manuscript; Q-FC and HL contributed to data analysis. All authors have read and approved the manuscript.

## Conflict of Interest Statement

The authors declare that the research was conducted in the absence of any commercial or financial relationships that could be construed as a potential conflict of interest.

## Footnotes

^1^www.encephalapp.com.

^2^http://www.rfmri.org/.

^3^http://www.fil.ion.ucl.ac.uk/spm.

^4^http://www.restfmri.net.

^5^http://rfmri.org/dpabi.

## Abbreviations

OHE, overt hepatic encephalopathy; HE, hepatic encephalopathy; ACC, anterior cingulate cortex; fMRI, functional magnetic resonance imaging; FC, functional connectivity; ECN, executive control network; DMN, default mode network; SN, salience network; RDLPFC, right dorsolateral prefrontal cortex; PCC, posterior cingulate cortex; RAI, right anterior insula; FDR, false discovery rate; MPFC, medial prefrontal cortex; AI, anterior insula; ICA, independent component analysis.
