# Supplementary material for: Disrupted Brain Intrinsic Networks and Executive Dysfunction in Cirrhotic Patients without Overt Hepatic Encephalopathy
Source: Front Neurol. 2018 Jan 25;9:14. doi: 10.3389/fneur.2018.00014 (PMC5788959; doi:10.3389/fneur.2018.00014)
Supplement: Supplementary file 1 [file Presentation_1.PDF]

Whether preprocessing should include global signal regression (GSR) is controversial; thus, we also conducted an analysis without the GSR, and the results were shown in the supplementary materials (Supplementary Figures 1–3).

Supplementary Figure 1 shows the FC maps for the ECN, DMN, and SN in the healthy controls and cirrhotic patients. The distribution of these 3 networks was similar with the results with GSR, but with the larger extent (Figure 1 *vs* Supplementary Figure 1).

Supplementary Figure 2 shows the mean network connectivity strengths for the 2 groups. Globally, the cirrhotic patients had significantly decreased connectivity in all 3 networks when compared with healthy controls. This finding without GSR was very consistent with the corresponding result with GSR (Figure 2 *vs* Supplementary Figure 2).

The voxel-wise analysis revealed several brain regions in which FC with the network seed was significantly reduced in cirrhotic patients; and no significant increase in FC was detected in any network (Supplementary Figure 3). Based on the analyses with and without the GSR, we obtained the patterns of between-group FC difference in the ECN, DMN, and SN; and we found that they were very similar to each other (Figure 3 *vs* Supplementary Figure 3).

Figure legends

Figure S1. Functional connectivity maps for healthy controls and cirrhotic patients, based on the analysis without the global signal regression in the fMRI data preprocessing. Yellow circles denote locations of the seed regions used to define each network. ECN, executive control network; DMN, default mode network; SN, salience network. A threshold of $P < 0.001$ (FDR corrected) was set to identify significance level.

Figure S2. Mean network connectivity strengths for the 2 groups. The cirrhotic patients showed significant reductions of mean functional connectivity strength within the executive control network (ECN), default mode network (DMN), and salience network (SN). This result was based on the analysis without the global signal regression in the fMRI data preprocessing.

Figure S3. Brain regions in which functional connectivity with the network seed was significantly reduced in the cirrhotic patients. ECN, executive control network; DMN, default mode network; SN, salience network. A threshold of $P < 0.05$ (FDR corrected) was set to identify significance level. This result was based on the analysis without the global signal regression in the fMRI data preprocessing.
