# Supplementary figures and images for: Disrupted Brain Intrinsic Networks and Executive Dysfunction in Cirrhotic Patients without Overt Hepatic Encephalopathy
Source: Front Neurol. 2018 Jan 25;9:14. doi: 10.3389/fneur.2018.00014 (PMC5788959; doi:10.3389/fneur.2018.00014)

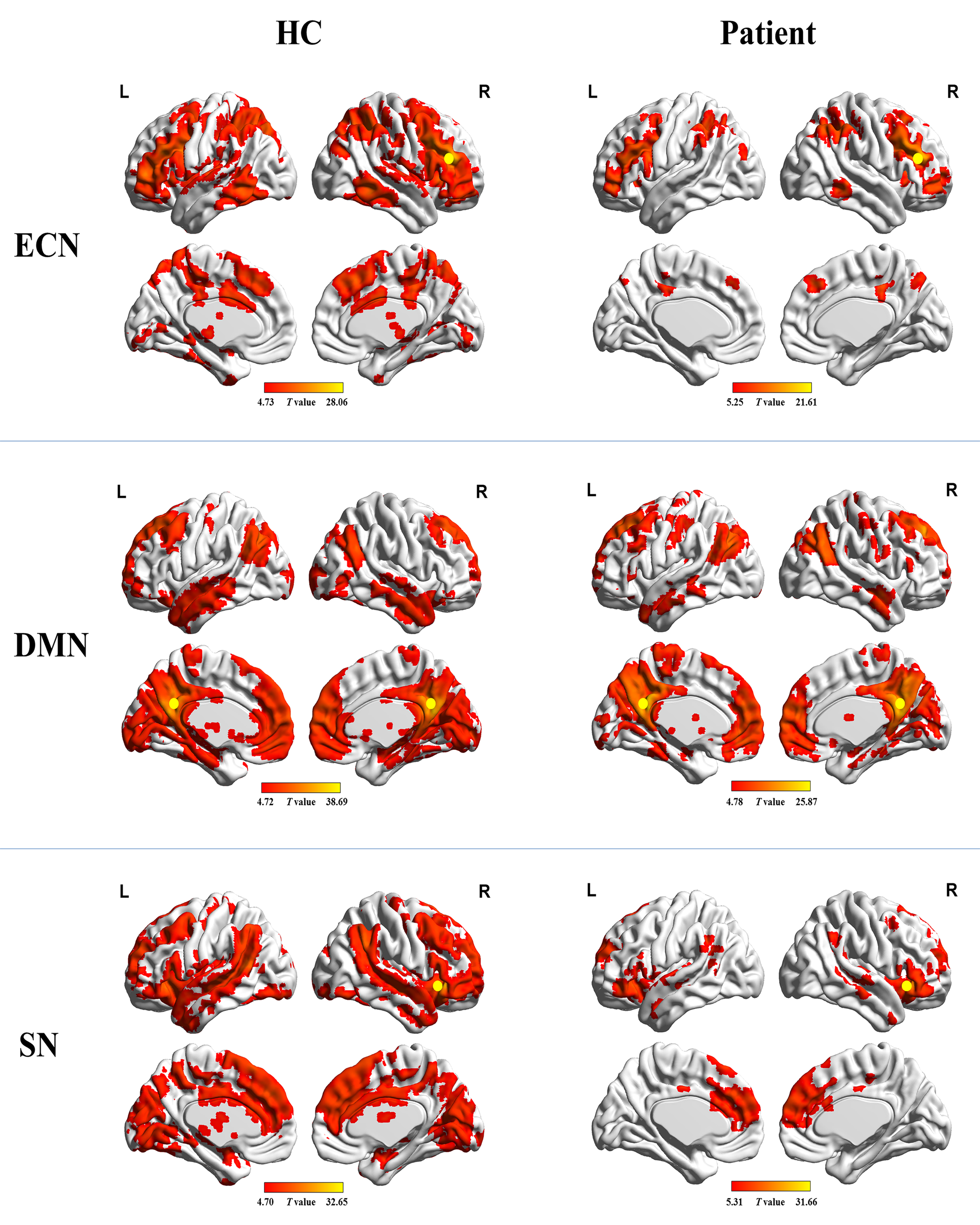

Supplement: Supplementary file 2 [file Image_1.TIF]

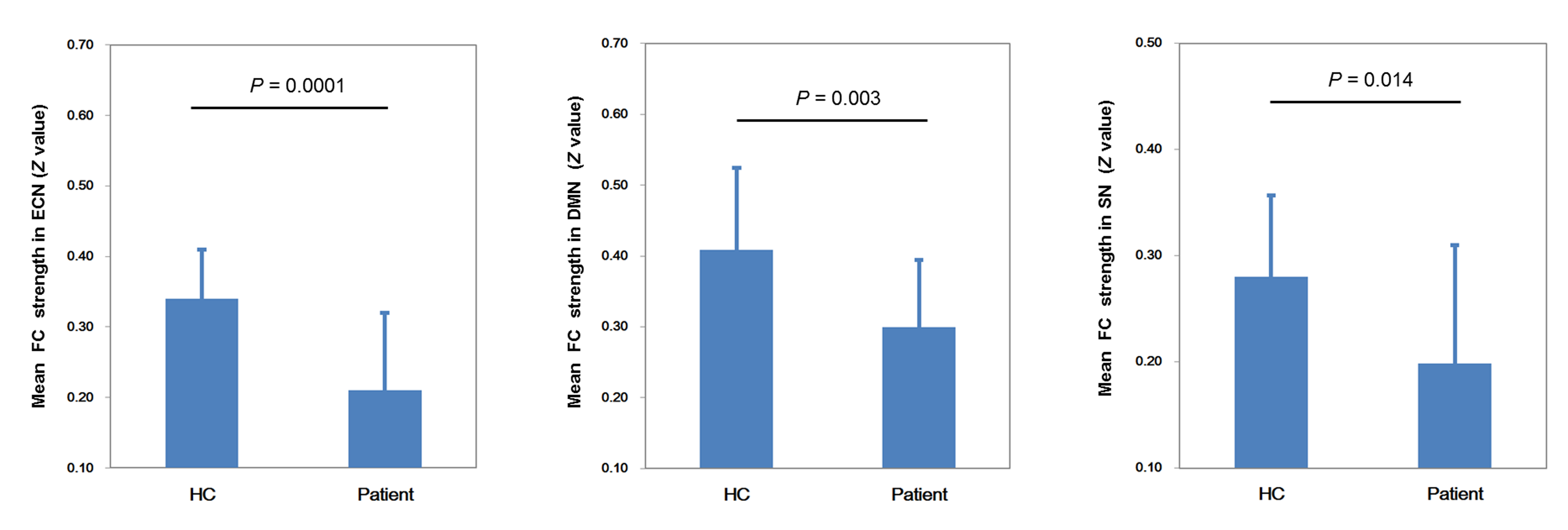

Supplement: Supplementary file 3 [file Image_2.TIF]

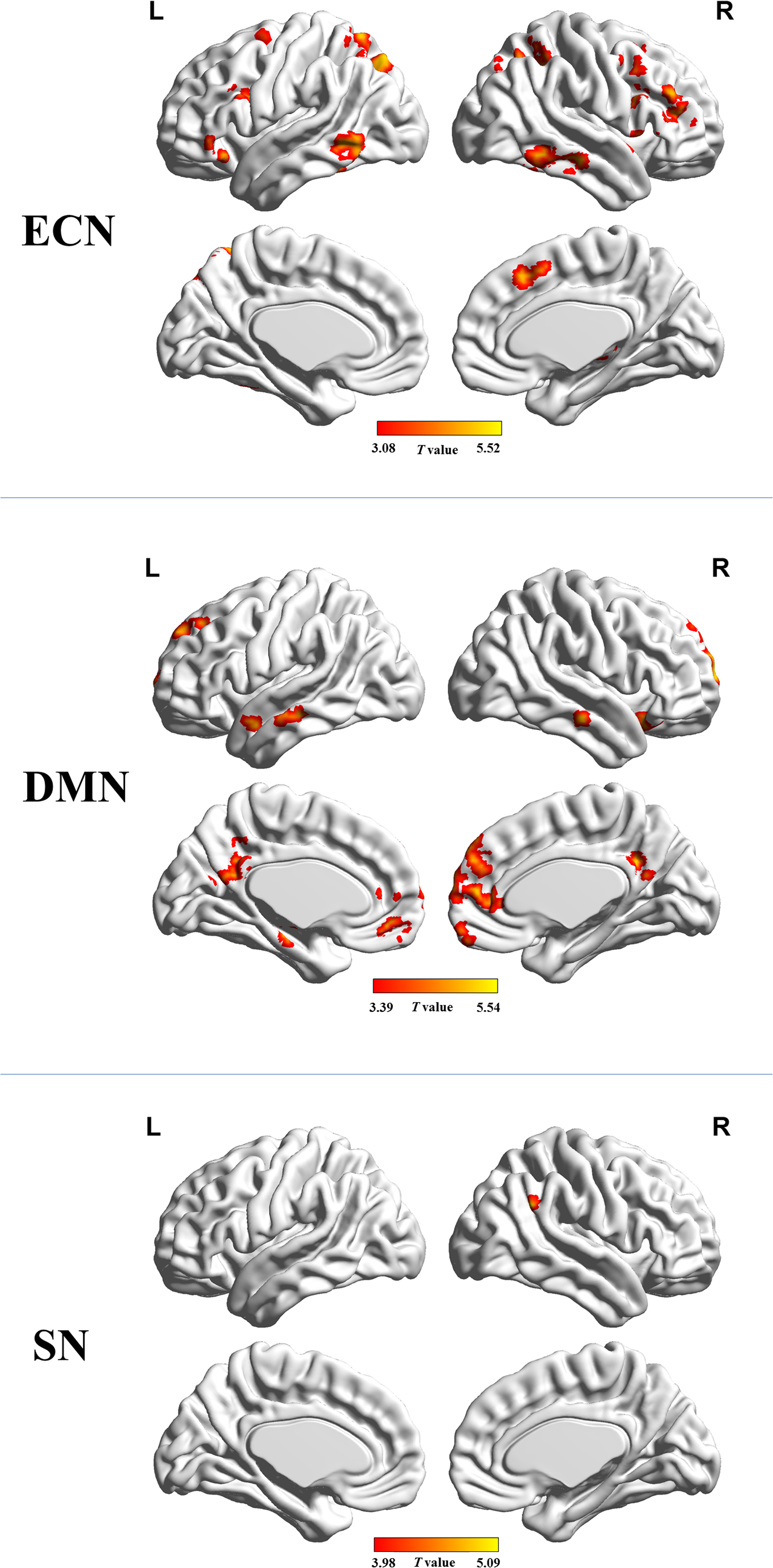

Supplement: Supplementary file 4 [file Image_3.TIF]
